# Individual differences in response to alcohol exposure in zebrafish (*Danio rerio*)

**DOI:** 10.1371/journal.pone.0198856

**Published:** 2018-06-07

**Authors:** Heloysa Araujo-Silva, Jaquelinne Pinheiro-da-Silva, Priscila F. Silva, Ana C. Luchiari

**Affiliations:** Department of Physiology, Bioscience Center, Federal University of Rio Grande do Norte, Natal, RN, Brazil; University of Leicester, UNITED KINGDOM

## Abstract

Personality traits are related to many aspects of one’s life, including risk taking, sociability, and behavioral changes caused by psychoactive substances. This study aimed to investigate individual differences and behavioral changes due to alcohol exposure in zebrafish (*Danio rerio*). To that end, adult animals were separated into two behavioral profiles: bold and shy, according to their risk taking behavior in an emergence test. Bold and shy fish were allowed to explore a 5-chamber tank and were tested for exploration and sociability (shoaling behavior) following alcohol exposure. The acute drug exposure treatments were 0.0%, 0.1% and 0.5% (v/v%) alcohol. The behavioral parameters evaluated were average and maximum swimming speed, total distance traveled, total time spent immobile, and time spent near a shoal or exploring the tank. For the groups that received no alcohol (0.0% alcohol), shy individuals spent more time near the shoal than bold fish. However, 0.5% alcohol increased bold fish responsiveness to the shoal, while both 0.1% and 0.5% alcohol diminished shoaling in shy fish. Our results show that the behavioral profiles of zebrafish are affected differently by alcohol, with shy animals seemingly more sensitive to behavioral change due to drug exposure. Moreover, we confirm zebrafish as a model for alcohol induced functional (exploration and social behavior) changes that could be useful in high throughput drug screens.

## Introduction

A number of studies have investigated the relationship between behavioral characteristics and alcohol consumption [1–4], albeit with contradictory results. The literature is limited and reports on addiction and behavioral profiles remain scarce. It is known that alcohol is the most widely consumed addictive substance in the world, and alcoholism is one of the most serious social problems [5,6]. While the types of treatment against alcoholism are still restricted and considered inefficient [7], abusive long-term consumption of alcoholic beverages causes serious adverse effects on tissue and brain function, ranging from memory lapses to complete dementia [8–11].

Characterizing the behavioral profile of an individual depends on a wide-ranging and varied behavioral as well physiological analysis, making it difficult to establish a relationship between profile and alcoholism. However, the increased propensity for some profiles to develop dependence requires further research [12,13]. Individual differences in behavior have long been ignored. However, in recent decades, the study of behavioral variability among individuals of the same species, the so-called temperament [14], personality [15] or behavioral syndrome [16], has been gaining ground because individuals exhibit different responses to similar environmental challenges [14,17,18]. It is known that individuals differ in their vulnerability to diseases. While some are resilient and others more susceptible [19], they also differ in the likelihood of developing disorders such as depression/anxiety after experiencing a traumatic event, or becoming addicted after using a particular drug [20]. The same pattern can be observed in other species. For instance, rats show differences in cocaine response when re-exposed to the drug after a period of abstinence, and only a small portion of the population exhibits seeking behavior [21].

Individual differences in the shy-bold dimension (boldness) demonstrate the propensity of an individual to take risks and explore novelty [22]. Individuals classified as bold are considered risk prone, and are thus expected to confront new situations and expose themselves more than shy individuals [23,24]. Bold individuals are also less attached to the social group, less anxious and less responsive to stress when compared to their shy counterparts [23]. Since alcohol is known to alter how one deals with the physical and social environment [25–28], bold and shy individuals would be expected to react differently when exposed to this drug. In this respect, the present study aimed to investigate the exploration and aggregation (social) behavior exhibited by bold and shy individuals under the influence of alcohol

According to Cavigelli [19], animal models can be extremely effective in elucidating the relationships between disease vulnerability and an individual's behavioral profile. In order for an animal to be considered an ideal model for translational research, at least 3 criteria must be met: face validation, predictive validation and constructive validation. Face validation refers to how representative of human behavior the animal model is; predictive validation is usually retroactive and requires the animal model to be manipulated in order to exert similar effects to those observed in humans; and constructive validation refers to the mechanisms by which the model fits the situations analyzed [29,30]. A number of alcohol studies [28,31–39] have tested zebrafish for these three principles. The present study evaluates individual differences and the effects of alcohol on zebrafish, one of the most widely used models in neuroethological research due to the easy application of genetic, embryological and behavioral methods [40,41]. This species has a complex behavioral repertoire, displays social preference for conspecifics, and shares more than 70% genetic homology with humans, constituting a highly translational model [41–43].

## Materials and methods

### Animals and housing

Adult zebrafish (n = 150, both sexes, wild-type) were obtained from a local farm (Natal, Rio Grande do Norte state) and housed in 50 L tanks with a multistage filtration system. Temperature, pH, and oxygen were measured regularly (maintained at 28°C, pH ~ 6.7, O2 ~ 6mg/L) and a 12h light/12h dark photoperiod was adopted. Fish were fed twice a day with commercial pelleted food (Alcon Guppy, 44% protein; 5% fat), live brine shrimp and frozen *Artemia salina*.

All the procedures were approved by the Animal Ethics Committee of the Federal University of Rio Grande do Norte (CEUA 041/2015).

### Behavioral profile determination

**Fig 1:**
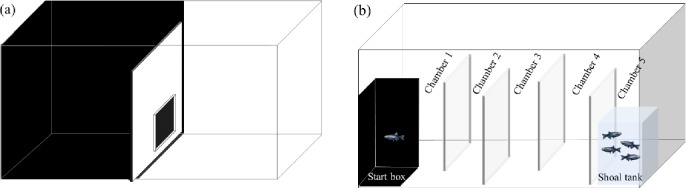
**Schematic overview of the (a) emergence tank and (b) exploration/sociability tank.** Tanks measured 40 x 25 x 20 cm (15L). For the emergence tank, an opaque glass wall with a 2 cm guillotine door allowed the fish to swim from the black to white compartment; all the surrounding walls were completely covered with opaque self-adhesive plastic films (white or black). Transparent walls divided the exploration/sociability tank into 5 chambers; the first chamber contained the ‘starting box’, and the fifth chamber ended in the ‘shoal tank’. Chambers 1, 2 and 3 were considered pathway areas. Chambers 4 and 5 were considered shoal areas, since these two chambers were bordering the stimulus tank.

Behavioral profiles were established according to the protocol described by Tudorache [44], which is based on the tendency of zebrafish to prefer dark areas and remain close to a group. After being housed for 15 days under the aforementioned conditions (stock), fish (n = 150) were randomly selected and divided into 15 groups of 10 individuals each. For the emergence test, the 15 L tank (40 cm x 25 cm x 20 cm, width x depth x height) was divided by an opaque partition into two equal-sized compartments: a dark side with black walls (initial zone) and a white compartment with white walls. In the middle of the partition, a guillotine door was used to control the passage of fish (Fig 1A). The fish group was placed in the initial zone (black) and allowed to acclimatize for 2 min. Next, the door was raised until the first individual passed through it; the door was then closed for 30 s and the fish transferred from the white compartment to another tank. The door was reopened until the next individual passed through it and then closed for 30 s, and so on. Fish were submitted to this procedure only once to avoid habituation and learning, which make it difficult to measure and affect animal response across trials, leading to variations in personality measurement. This procedure was repeated for all animals, until a stock of 45 bold (the first three individuals moving from the dark compartment to the white in every 10-fish group) and 45 shy fish (the last three to leave the initial zone in every 10-fish group) was obtained. Intermediate animals were excluded from the tests and returned to the stock tanks.

Six groups of fish were formed: three of bold fish (n = 15/ each group) and three of shy fish (n = 15/ each group), with each group housed in a different glass home tank. Fish were then submitted to an habituation phase in a 5-chamber tank (14 days, one trial per day), and on the 15^th^ day, were exposed to alcohol and tested in the same tank, as follows.

### Exploration and sociability test

For this test we used a tank (40 cm× 25 cm × 20 cm, Fig 1B) divided into 5 chambers. All walls were transparent so that fish could see the whole tank from any vantage point. The starting area was a small (5 cm x 5 cm) black box with a guillotine door.

During the habituation phase, each fish was individually transferred from its respective home tank to the black starting area of the 5-chamber tank with the lift-up partition closed. After 60 s acclimation, the opaque partition was raised 2 cm and the fish had up to 10 min to freely navigate through the test tank and swim between chambers. The prolonged habituation phase allowed fish to decrease stress associated with novelty and social isolation.

On the 15^th^ day (probe day), fish were individually transferred from their respective home tank to a 2L tank for acute alcohol exposure, where they were kept for 60 min. For alcohol exposure, 99.9% absolute ethyl alcohol (Dinâmica, Química contemporânea Ltda. Brazil) was used to prepare two dilutions with system water: 0.1% alcohol and 0.5% alcohol. Only system water was used for the 0.0% alcohol. Thus, different alcohol concentrations and behavioral profiles could be tested in the six groups (3 bold and 3 shy): Bold 0.0% alcohol (n = 15), Shy 0.0% alcohol (n = 15), Bold 0.1% alcohol (n = 15), Shy 0.1% alcohol (n = 15), Bold 0.5% alcohol (n = 15), and Shy 0.5% alcohol (n = 15). Each group was submitted to the exploration and sociability tests described above.

Immediately after alcohol exposure, fish were transferred to the starting area of the 5-chamber tank for 60 s acclimation. However, only on probe day, a small transparent tank was placed in the last chamber, into which a shoal of 4 conspecific zebrafish (same size as the experimental fish) was placed as social stimulus. After 60 s in the starting area, fish were allowed to freely explore the tank and interact with the conspecifics for 10 min.

Fish behavior was recorded for 10 min, using a handcam installed 50 cm above the tank. Behavior was analyzed by tracking software (ZebTrack / UFRN) [45] developed in the MATLAB platform (R2014a, Math Works, Natick, MA). The parameters evaluated were time spent near the shoal or exploring the other tank chambers, total distance traveled, average swimming speed, maximum swimming speed, and total time spent immobile at some point in the tank (average time not moving through the chambers).

### Statistical analysis

After initial data analyses for normality and homoscedasticity, we conducted two-way ANOVA (followed by the Student-Newman-Keuls post hoc test) to test intergroup differences in average swimming speed, maximum swimming speed, total distance traveled, and total time spent immobile. The time spent in each chamber of the tank was divided into two categories: ‘pathway areas’ (chambers 1, 2 and 3), the areas fish had to swim to reach the shoal, and ‘shoal areas’ (chambers 4 and 5), the two chambers in which fish could access the shoal (Fig 1B). Next, we used two-way ANOVA (followed by the Student-Newman-Keuls post hoc test) to test intergroup differences in their profile (bold or shy) and alcohol treatment (0.0, 0.1, and 0.5%), and the paired Student’s t-test to evaluate differences in time spent in the two areas (pathways vs. shoal areas) for each profile and treatment group. In all cases, statistical significance was set at α <0.05.

## Results

**Fig 2:**
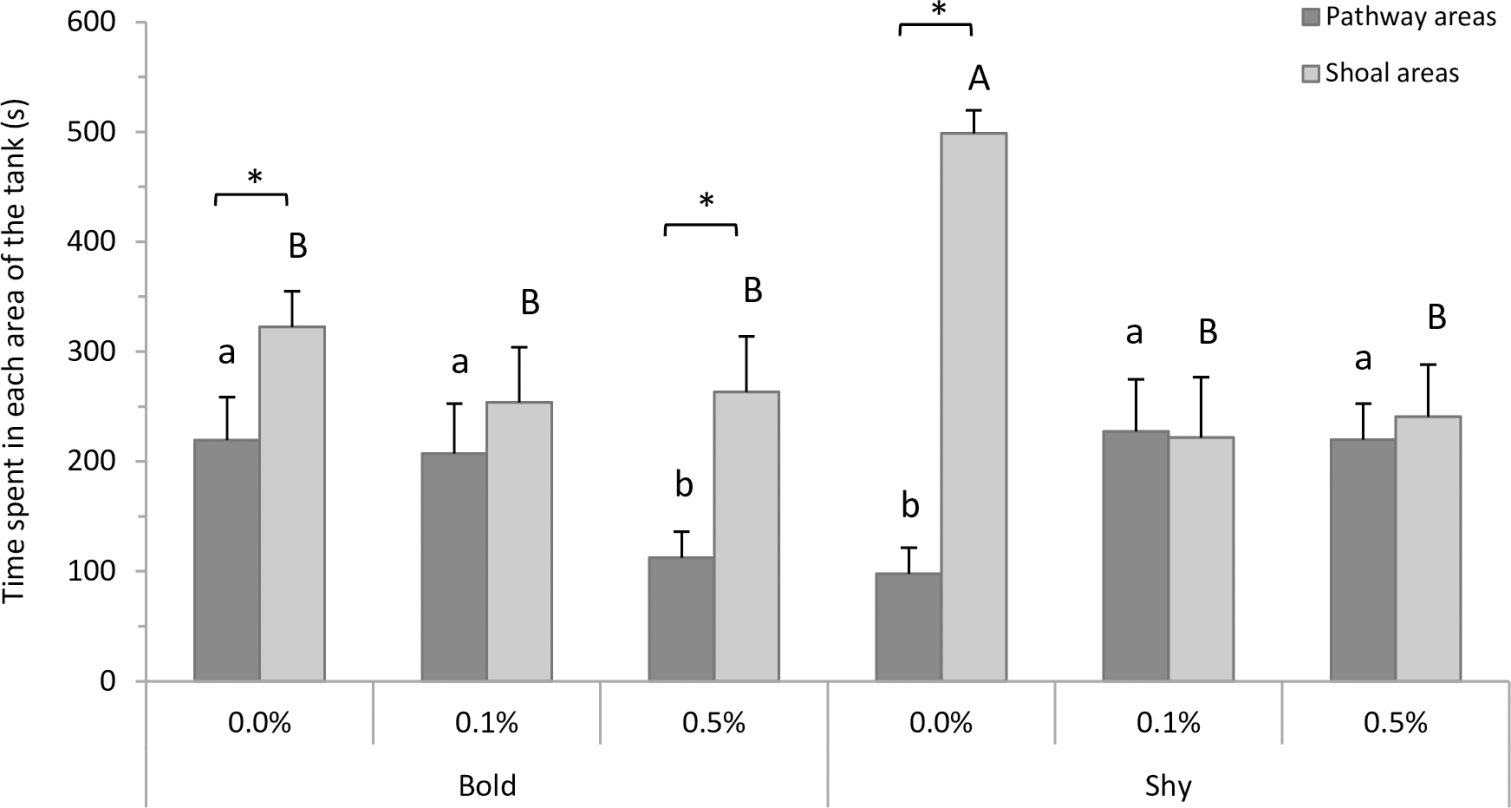
Time zebrafish spent in each area of the tank (mean + SEM) during the exploration/sociability test. Different lowercase letters indicate statistical significance between pathways areas (bold and shy groups: two-way ANOVA, p< 0.05). Different capital letters indicate statistical significance among shoal areas (bold and shy groups: two-way ANOVA, p<0.05. Asterisk indicates a statistical difference between pathway and shoal areas, in the same alcohol treatment (p<0.05).

Fig 2 depicts the time spent in the pathway (chambers 1, 2 and 3) and shoal areas (chambers 4 and 5) for zebrafish categorized as bold and shy, and exposed to 0.0, 0.1 or 0.5% alcohol. Time spent in tank areas of interest varied between groups because the animals’ latency to leave the starting box was not considered for this data set (some fish stayed in the starting area for longer). For the pathway areas, two-way ANOVA revealed no significant alcohol treatment (F_2,78_ = 1.51 p = 0.23) or profile effect (F_1,78_ = 0.004 p = 0.94), but the interaction between alcohol treatment and profile was statistically significant (F_2,78_ = 4.98 p = 0.009). The Student-Newman-Keuls test showed that the bold 0.5% and shy 0.0% groups spent less time in pathway areas than the other four groups (p<0.05). For the shoal areas, two-way ANOVA indicated no significant profile effect (F_1,78_ = 1.25 p = 0.26), but significant alcohol treatment (F_2,78_ = 9.38 p<0.001) and alcohol treatment vs. profile interaction effects (F_2,78_ = 3.52 p = 0.03). The Student-Newman-Keuls test revealed that the shy 0.0% alcohol group spent more time in the shoal areas than all the other groups (p<0.05).

The Student’s t-test, used time in the pathway and shoal areas for each group, showed that the bold 0.0% alcohol (t = -2.04 p = 0.05), shy 0.0% alcohol (t = -12.74 p<0.01) and bold 0.5% alcohol groups (t = -2.69 p = 0.01) spent significantly more time in the shoal than the pathway area, while bold 0.1% alcohol (t = -0.69 p = 0.49), shy 0.1% alcohol (t = 0.07 p = 0.94) and shy 0.5% alcohol (t = -0.36 p = 0.72) were not statistically significant (Fig 2).

**Fig 3:**
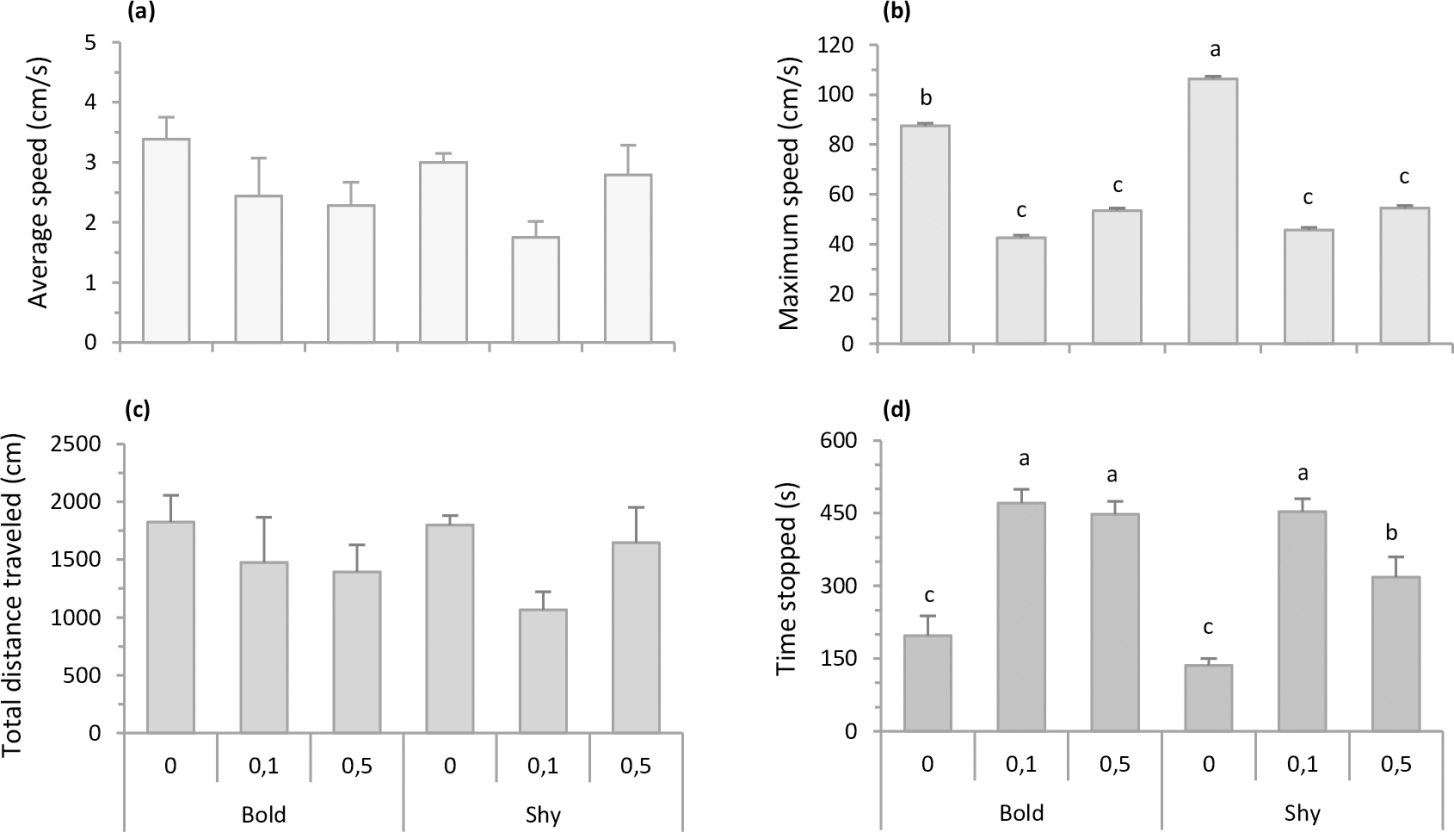
Comparison between bold and shy experimental groups following alcohol exposure. Bars indicates zebrafish locomotor behavior (mean + SEM) for 10 min in the test tank: (a) Average swimming speed, (b) Maximum swimming speed, (c) Total distance traveled, and (d) Total time spent immobile. Average speed and total distance traveled were not statistically significant between treatments. Different letters indicate statistical significance between alcohol treatments (two-ay ANOVA, p<0.05).

Fig 3 shows the statistical significance between the bold and shy profile under the effect of 0.0%, 0.1%, and 0.5% alcohol exposure, with respect to average swimming speed, maximum swimming speed, total distance traveled, and total time spent immobile. Two-way ANOVA demonstrated that profile and alcohol exposure did not affect average speed (profile F_1,78_ = 2.60 p = 0.06; alcohol treatment F_2,78_ = 0.32 p = 0.57; profile vs. alcohol treatment F_2,78_ = 1.13 p = 0.32; Fig 3A) or total distance traveled (profile F_1,78_ = 0.08 p = 0.10; alcohol treatment F_2,78_ = 2.28 p = 0.10; and profile vs. alcohol treatment F_2,78_ = 0.86 p = 0.42; Fig 3C). For maximum swimming speed, two-way ANOVA indicated a significant alcohol treatment effect (F_2,78_ = 35.64 p<0.01), but no significant profile (F_1,78_ = 1.96 p = 0.16) or profile vs. alcohol treatment interaction effect (F_2,78_ = 1.07 p = 0.34; Fig 3B). The Student-Newman-Keuls test showed higher maximum speed in bold 0.0% alcohol and shy 0.0% alcohol than the other groups (p<0.05; Fig 3B). For total time spent immobile, two-way ANOVA revealed a significant alcohol treatment (F_2,78_ = 48.15 p<0.01) and profile treatment effect (F_1,78_ = 7.69 p = 0.007), but no statistical profile vs. alcohol treatment interaction effect (F_2,78_ = 1.55 p = 0.22; Fig 3D). The Student-Newman-Keuls test showed that bold 0.0% and shy 0.0% individuals spent less time immobile than the other four groups (p<0.05; Fig 3D).

All data of each variable sample in this study are available as supporting information (S1 File).

## Discussion

In the present study we observed the effects of alcohol exposure on exploration and social behavior in two zebrafish profiles: bold and shy. In both the laboratory and in nature, zebrafish display strong social behavior, recognized as aggregation, group formation or shoaling [46,47]. For this and other species, social behavior has high adaptive significance, such as increasing reproductive success, avoiding predators and improving foraging success [48–50]. Our results suggest that social and exploratory differences seem to be related to individual behavioral profiles; bold individuals explore more and are less inclined to shoal with a group, while shy zebrafish spend significantly more time shoaling than exploring the environment, corroborating other findings [51–53]. This response might be related to the value each animal attributes to the resource (social group), that is, social animals consider the shoal to be more valuable than less social animals.

Additionally, psychotropic drugs such as alcohol alter the way an animal perceives the environment and ranks a resource. Shy animals were affected by both 0.1% and 0.5% alcohol and decreased shoaling under its effects, but bold animals changed their behavior only when exposed to 0.5% alcohol, suggesting that sensitivity to alcohol has different effects on animal profiles.

The bold-shy continuum has been widely investigated and is characterized mainly based on the individual tendency to take risks [14]. Bold individuals are risk prone, more active and more aggressive, and cope better with social isolation. By contrast, shy individuals are risk averse, less aggressive, explore less, and are more dependent on the social group [51–53]. The results obtained here for bold and shy zebrafish exposed to 0.0% alcohol (control) corroborate this characterization. In fact, zebrafish are a highly exploratory and social species that prefer to be close to their conspecifics [54]. Numerous studies show that novelty prompts increased exploration in zebrafish [55–59]. This is adaptive behavior, since animals may find new sources of food, shelter and flight routes by better exploring all new features of the environment [56]. In this study, we used social novelty to test fish behavior. Irrespective of the profile, zebrafish responded to the social group by exploring and interacting (visually) with the shoal. However, bold animals continued to explore the tank after locating the shoal, while shy animals remained close to it for a longer period of time (Fig 2). According to Roy [60], leaving the shoal is a strong measure of boldness. Since shoaling is a common defensive behavior in zebrafish [61], risk-averse individuals (i.e. shy) are expected to adopt this response more frequently, exhibiting a tendency to stay close to the group.

Moreover, out study shows that bold and shy zebrafish displayed significant differences in exploratory and social behavior. This is the first time data on the effects of alcohol on bold and shy profiles have been presented. Our results indicate that alcohol exposure alters profile behavior. Shy fish exposed to 0.1% and 0.5% alcohol exhibited less shoaling compared to the control group (shy 0.0% alcohol), suggesting that alcohol decreased risk aversion behavior and changed the resource value attributed by shy zebrafish. This social response switch may be related to anxiety-like behavior. Indeed, acute alcohol exposure acts as a potent anxiolytic [62]. When alcohol in the bloodstream penetrates the brain, it stimulates neurons to release extra serotonin and dopamine, neurotransmitters that regulate pleasure, mood, and anxiety [63]. As a result, one of the first effects of alcohol is to increase disinhibition and euphoria. Additionally, this substance can affect two other neurotransmitters by inhibiting the glutamatergic system and stimulating the GABAergic system [64], initially inducing a calming effect. Other studies have shown that low alcohol doses decrease bottom-dwelling and freezing, both anxiety indicators, while high doses raise anxiety-like behaviors [65]. Even though we did not evaluate anxiety-related parameters, the changes observed in shy fish behavior seem to be related to decreased anxiety due to alcohol exposure.

In recent years, individual differences in behavior and physiology have been the focus of research. With respect to the bold-shy aspect, behavior has been characterized, and physiology seems to corroborate the behavioral repertory. Bold individuals, also classified as proactive, exhibit higher basal cortisol than their shy counterparts, which are classified as reactive [20,52,66]. However, stressors cause lower HPI (hypothalamus-pituitary-interrenal) response in bold than in shy fish [20,52,66,67]. In terms of neurotransmitters, lower brain serotonergic activity and higher dopaminergic activity characterize proactive (bold) individuals, while reactive animals (shy) display higher serotonin and lower dopamine levels, which may also contribute to the behavioral differences in these profiles [20,52,66,68,69]. In this respect, we believe psychoactive drugs have a different effect on physiology and behavioral profiles. For instance, addictive drugs, such as alcohol, induce an increase in dopamine transmission, which may be related to the initial feeling of pleasure and subsequent addiction [70].

In the present study, shy and bold zebrafish showed opposite responses to alcohol, implying that inherent individual characteristics are related to this finding. Individual vulnerability to alcohol depends on several factors, including genetic predisposition, environmental characteristics and life history [71]. Shy animals exposed to 0.1% and 0.5% alcohol explored more and decreased shoaling time, which seems to be related to decreased anxiety and dependence on the group, a feature associated with the bold profile. We suggest that alcohol may have increased dopaminergic transmission in these animals, inducing a state of increased euphoria. This response deserves careful investigation because dopamine is one of the elements related to addiction and the behavioral change observed in the shy animals may reflect their higher propensity to drug seeking and dependence.

In contrast to the changes observed in shy fish under the effects of alcohol, the bold fish became more social and less exploratory when exposed to 0.5% alcohol. It seems 0.1% alcohol did not affect bold fish behavior, but increased doses may have influenced both serotonergic and dopaminergic transmission in these animals, and the serotonin increase could be related to the considerable social interaction exhibited by the bold 0.5% alcohol group. Dzieweczynski et al. [72] showed that serotonin exposure decreases boldness in fish and, as such, we speculated that 0.5% alcohol increased serotonin levels, which reduced risk taking behavior (decreased exploration) and increased sociability. However, future studies investigating the effects of different alcohol treatments on brain serotonin and dopamine levels in bold and shy zebrafish are needed to confirm this hypothesis.

It is well established that alcohol exposure induces dose-dependent neurological and psychological effects. A low to moderate concentration produces stimulating and anxiolytic effects, while higher concentrations lead to loss of motor control, disorientation and sedation due to the depressive effect of alcohol [39,73–75]. In this respect, one could argue that alcohol exposure may have led to exploratory/social behavior differences due to locomotor stimulation or impairment. This hypothesis can likely be rejected in the present study because the alcohol concentrations applied here did not cause changes in average speed or distance traveled (Fig 3A and 3C). Alcohol exposure at 0.1 and 0.5% seems to have decreased maximum swimming speed and increased time spent immobile, regardless of fish profile (Fig 3B and 3D), which could be related to the anxiolytic effects of low alcohol concentrations. In this regard, the effects of alcohol, along with the prolonged habituation phase, decreased fish stress and may have contributed to profile performance in terms of exhibiting the authentic effects on bold and shy profiles.

Our results corroborate the occurrence of individual differences in zebrafish [51,60,76] and suggest varying susceptibility to alcohol depending on the behavioral profile. It is important to point out that these differences may be related to aggressive behavior and anxiety, parameters not assessed in our study, but worthy of future investigation. In addition, the resource used here (social group) had a significant impact on fish response. For instance, the reduced time bold fish spent with the shoal compared to shy fish did not represent impaired ability in locating the group in the tank, but rather lower the shoaling interest inherent to this profile. Thus, testing other types of stimuli, such as food or predator risk, may provide an alternative perspective to explain the differences in bold and shy profiles exposed to alcohol. However, the behavioral changes observed here were significant and represent an important step towards a thorough understanding of the effects of alcohol on different individuals.

The zebrafish is a popular model in the study of psychiatric diseases, such as alcohol abuse, its mechanisms and treatments [77]. Alcoholism is a multifactorial disorder, and the challenges faced by researchers include the varying vulnerability to addiction and treatment response among patients. As such, it is important to consider individual profiles as an influencing factor. Understanding the differences between individual profiles in a population is essential to elucidating how animals cope with their surroundings and how their decisions impact fitness, survival and evolution. Thus, our results add to the body of literature on the effects of alcohol, underscore an important feature that should be considered in drug abuse, and reinforce the suitability of zebrafish as a translational model in the study of complex human disorders.

## Supporting information

S1 File Data set of bold and shy zebrafish exposed to different acute alcohol treatments. Values presented show Average swimming speed, Maximum swimming speed, Total distance traveled, Total time spent immobile, and Time spent in the pathway (chambers 1, 2, 3) and shoal (chambers 4, 5) areas for each group.
(XLSX)
